# Supplementary material for: Disentangling the aging gene expression network of termite queens
Source: BMC Genomics. 2021 May 11;22:339. doi: 10.1186/s12864-021-07649-4 (PMC8114706; doi:10.1186/s12864-021-07649-4)
Supplement: Supplementary file 1 — Additional file 1: Figure S1. Schematic workflow. Details are explained in the Methods. Figure S2. WGCNA modules of co-expressed transcripts that negatively correlate with age. Modules are named after colors by WGCNA. Eigengenes of all these modules showed a negative correlation with age while * indicates that the age-correlation was significant. Modules marked with $ were found in the young subnetwork. Figure S3. WGCNA modules of co-expressed transcripts that positively correlate with age. Modules are named after colors by WGCNA. Eigengenes of all these modules showed a positive correlation with age; * indicates that the age-correlation was significant.Additionally, modules marked with $ were found in the old subnetwork. Figure S4. GO enrichment for the WGCNAmodule ‘tan’ that positively correlated with age and which had many old-age DETs. Details are shown for BP (Biological Process), which revealed an enrichment of transcripts for ribosomal and tRNA related functions. Figure S5. Young and old transcript subnetworks corresponding to the second level neighborhood of Iso-MaSigPro DETs. Age-related DETs were located in the WGCNA co-expression network and these DETs and their one- and two-step neighbors (i.e., the ‘second level neighborhood’) were then extracted from the co-expression network to provide the shown networks. Figure S6. BiNGO GO enrichment(Biological Process) for the young subnetwork. No terms were significantly enriched after correcting for multiple testing (FDR). Figure S7. BiNGO GO enrichment (Biological Process) for the old subnetwork. Colored nodes are GO terms that were significantly enriched after correcting for multiple testing (FDR). FigureS8. BiNGO GO enrichment of (Molecular Function; Cellular Component) for the old subnetwork. Colored nodes are GO terms that were significantly enriched after correcting for multiple testing (FDR). Figure S9. BiNGO GO enrichment (Biological Process; MolecularFunction; Cellular Component) for the ‘green’ WGCNA mo [file 12864_2021_7649_MOESM1_ESM.pdf]

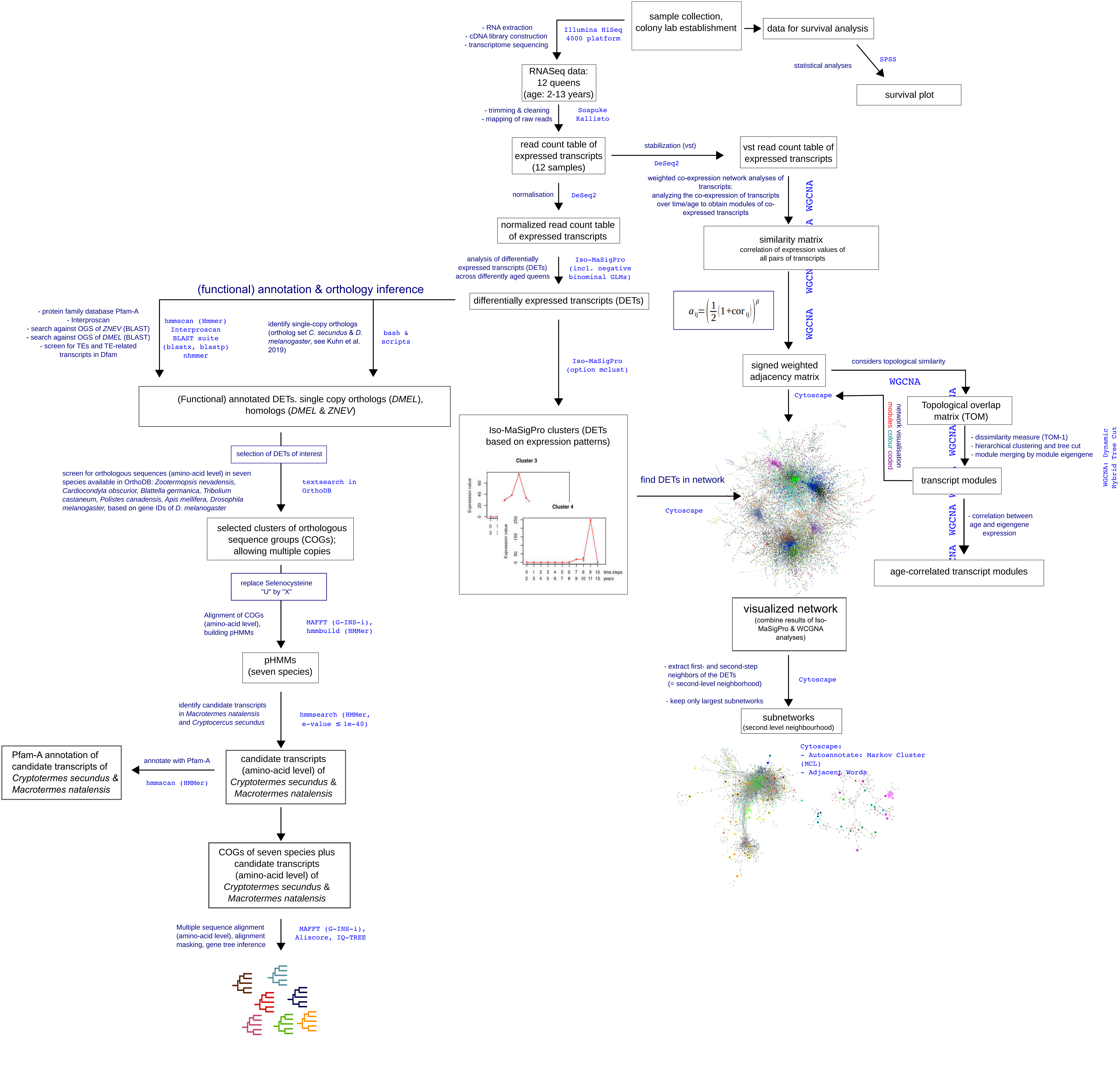

Figure S1: Schematic workflow.
Details are explained in the Methods.

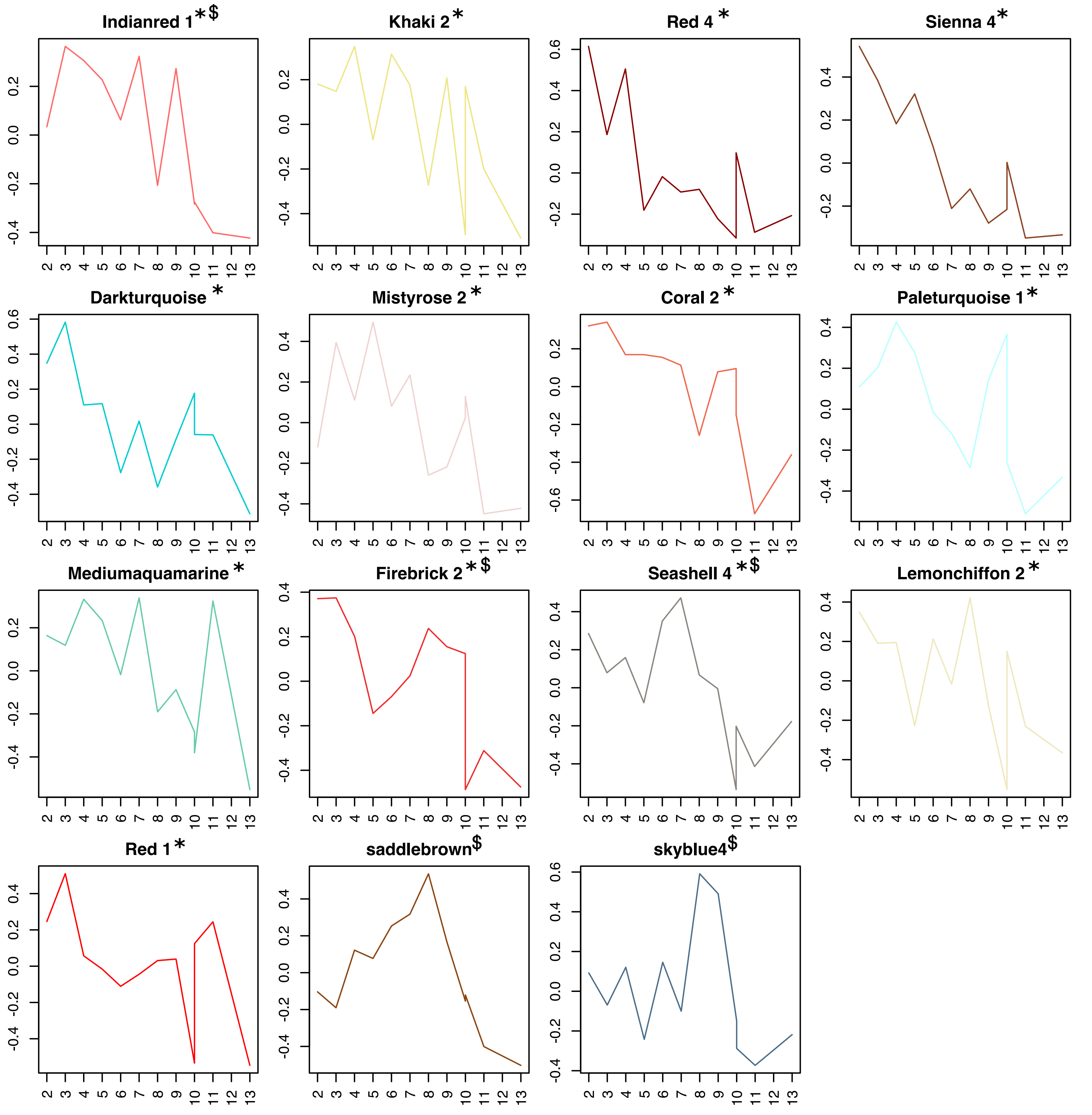

Figure S2: WGCNA modules of co-expressed transcripts that negatively correlate with age.
Modules are named after colors by WGCNA. Eigengenes of all these modules showed a negative correlation with age while * indicates that the age-correlation was significant. Modules marked with $ were found in the young subnetwork.

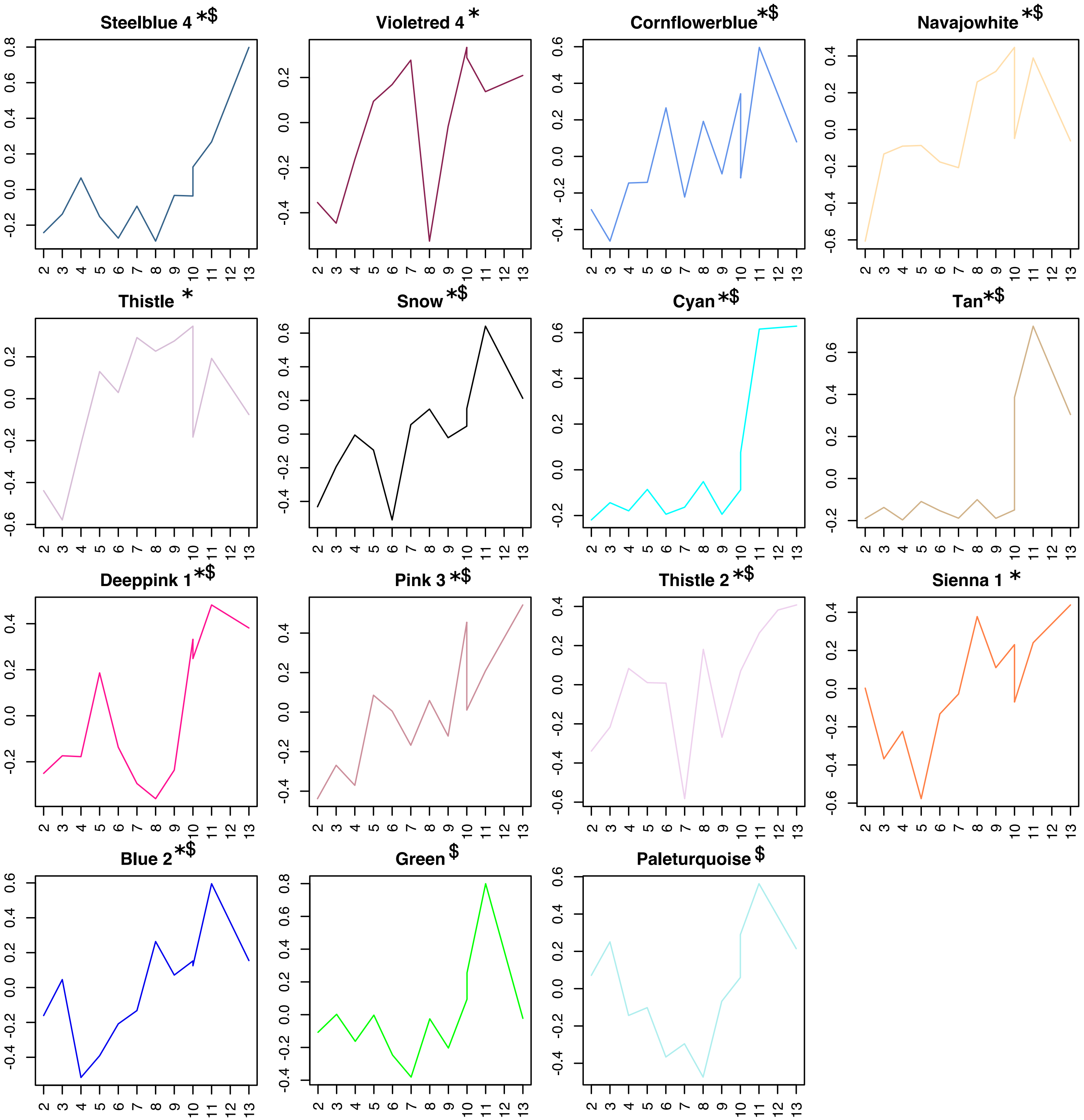

Figure S3: WGCNA modules of co-expressed transcripts that positively correlate with age. Modules are named after colors by WGCNA. Eigengenes of all these modules showed a positive correlation with age; * indicates that the age-correlation was significant. Additionally, modules marked with $ were found in the old subnetwork.

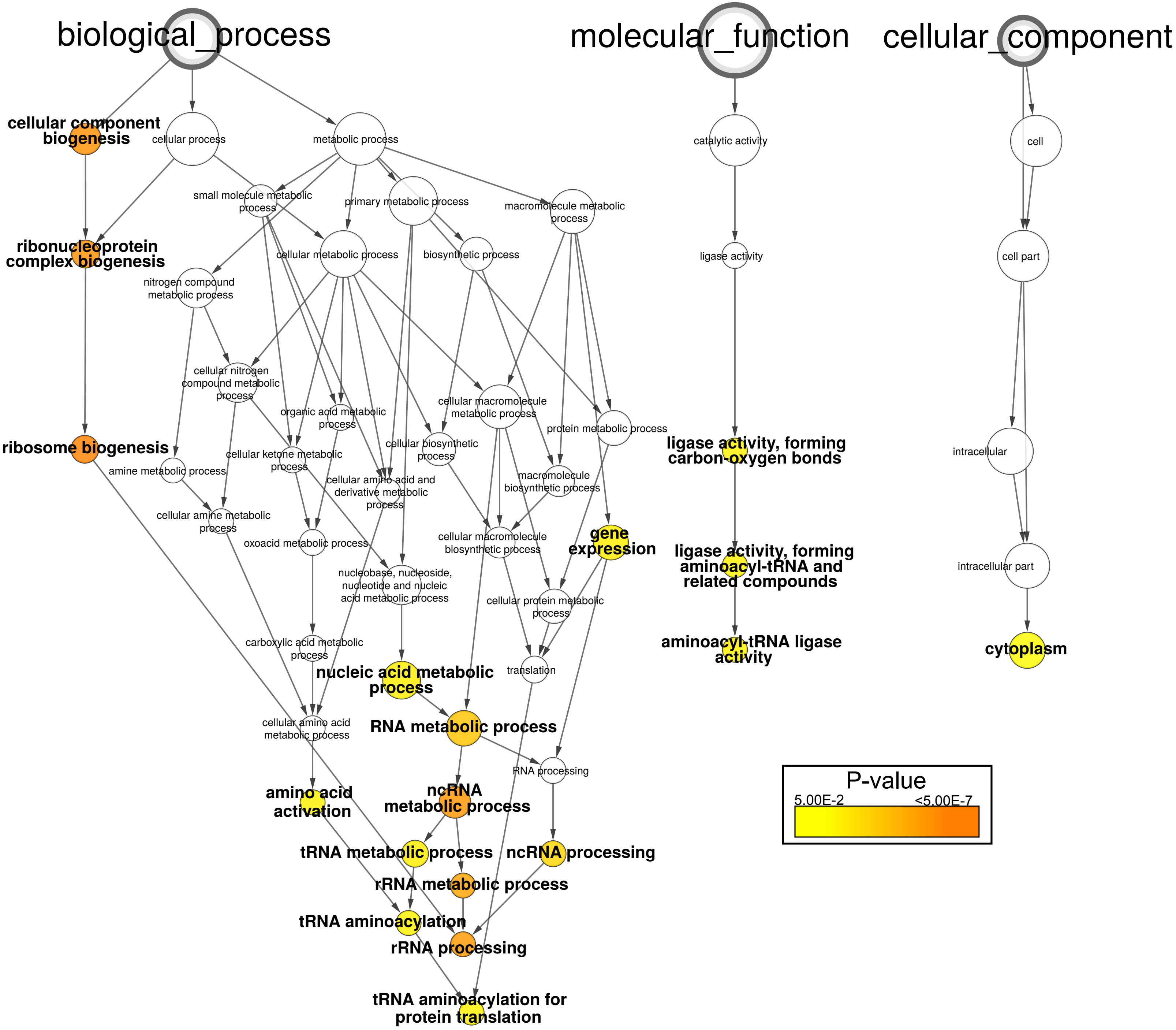

Figure S4: GO enrichment for the WGCNA module ‘tan’ that positively correlated with age and which had many old-age DETs.
Details are shown for BP (Biological Process), which revealed an enrichment of transcripts for ribosomal and tRNA related functions.

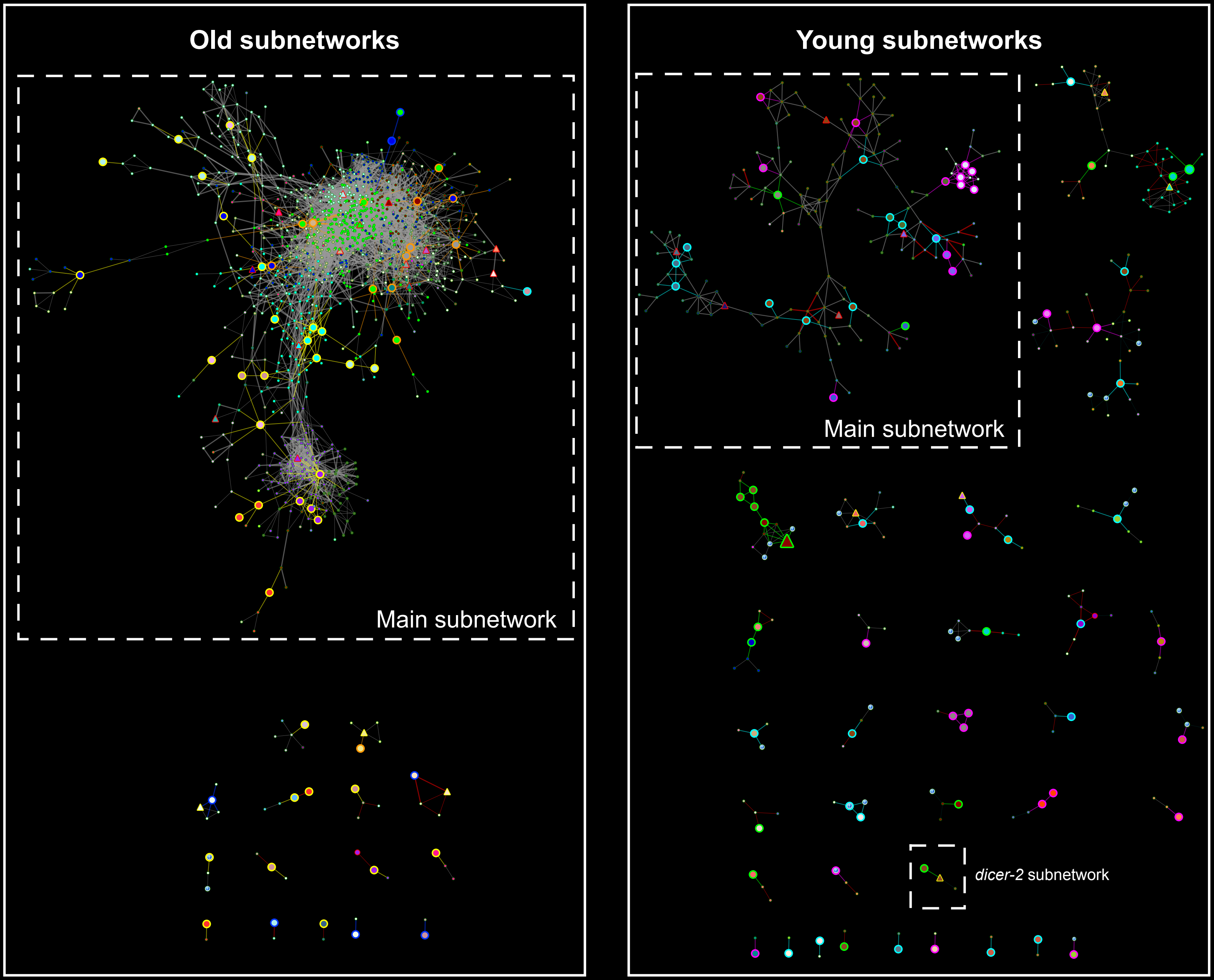

Figure S5: Young and old transcript subnetworks corresponding to the second level neighborhood of Iso-MaSigPro DETs.

Age-related DETs were located in the WGCNA co-expression network and these DETs and their one- and two-step neighbors (*i.e.*, the 'second level neighborhood') were then extracted from the co-expression network to provide the shown networks.

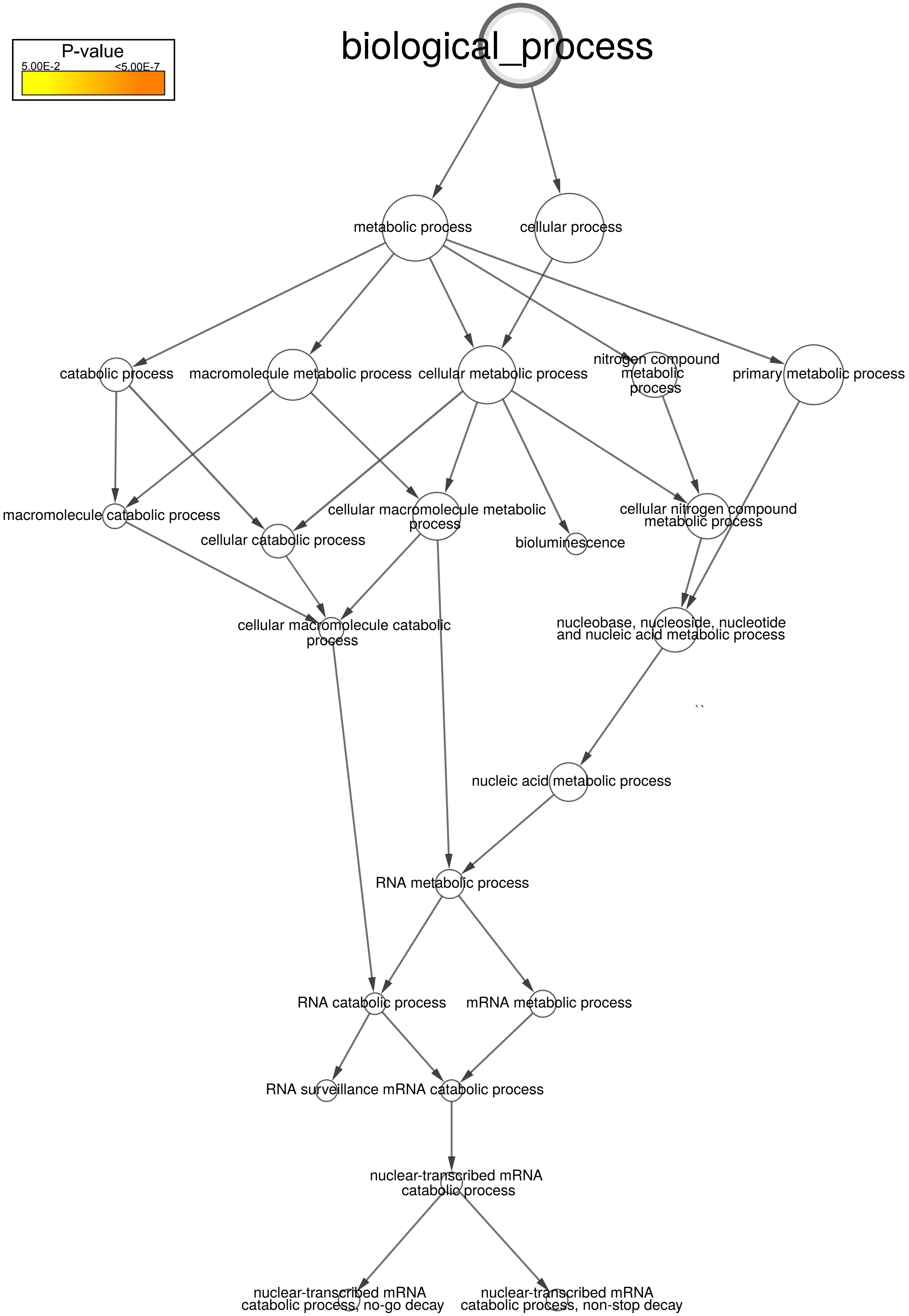

Figure S6: BiNGO GO enrichment (Biological Process) for the young subnetwork.
No terms were significantly enriched after correcting for multiple testing (FDR).

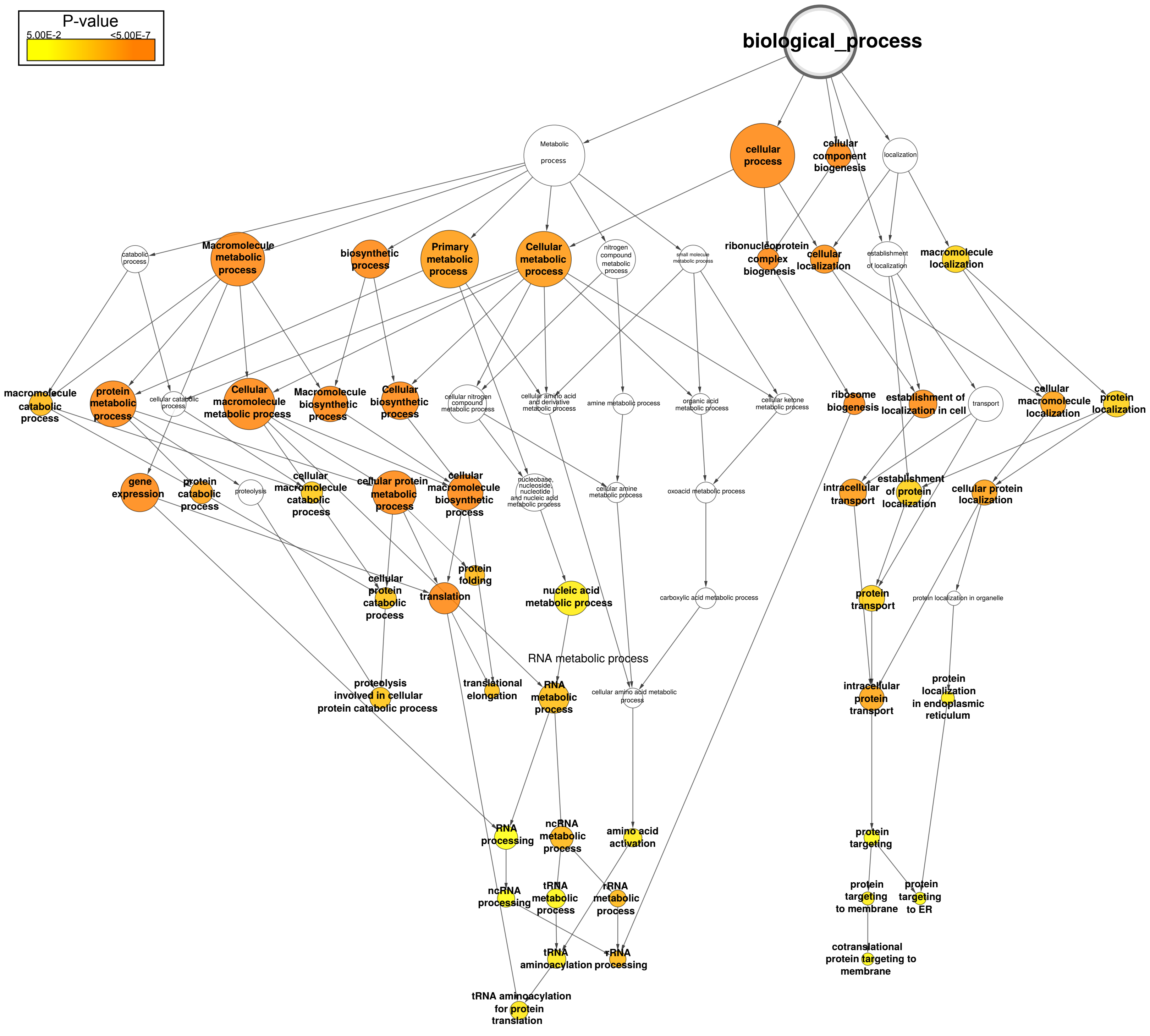

Figure S7: BiNGO GO enrichment (Biological Process) for the old subnetwork.
Colored nodes are GO terms that were significantly enriched after correcting for multiple testing (FDR).

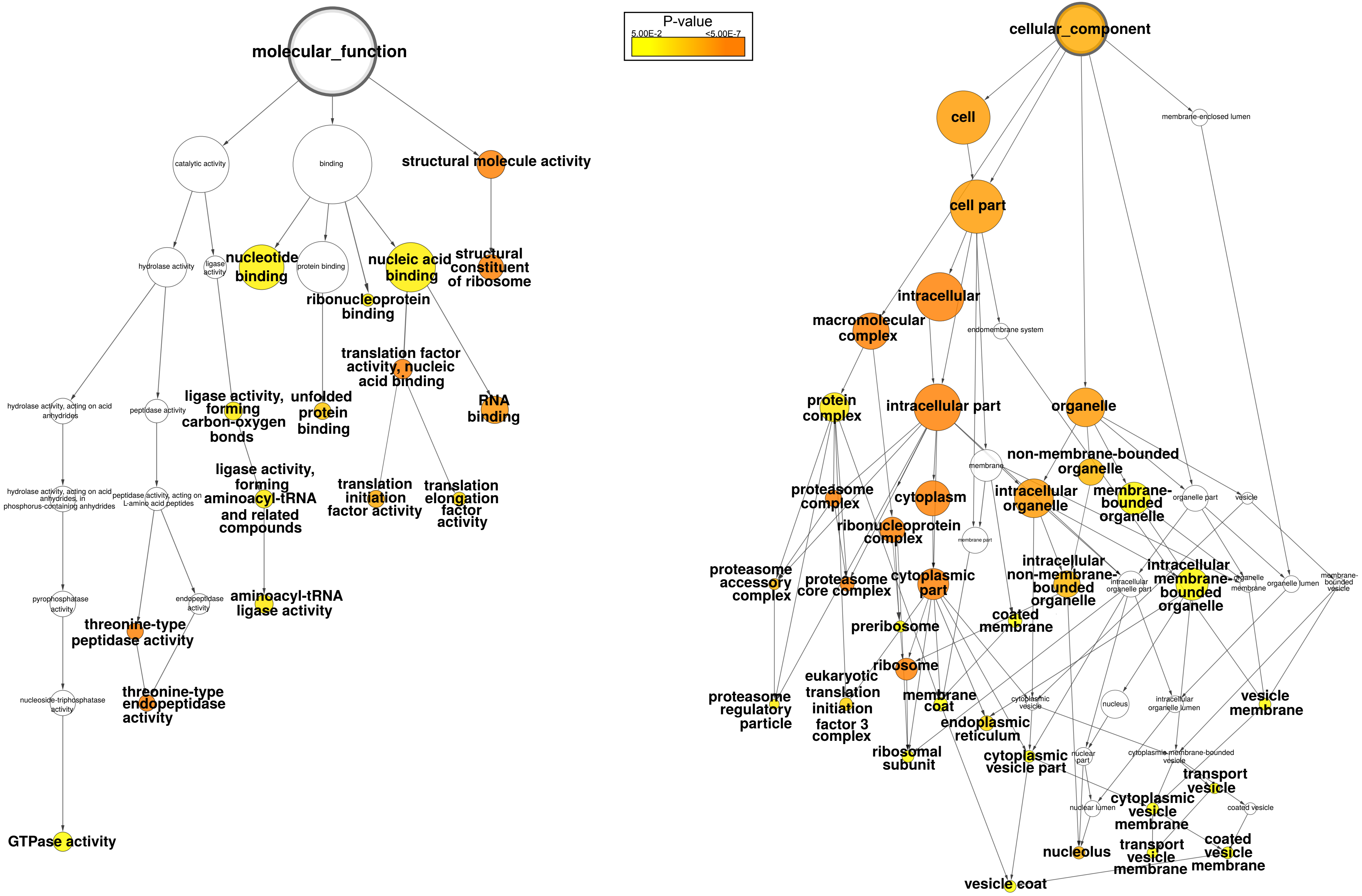

Figure S8: BiNGO GO enrichment of (Molecular Function; Cellular Component) for the old subnetwork.
Colored nodes are GO terms that were significantly enriched after correcting for multiple testing (FDR).

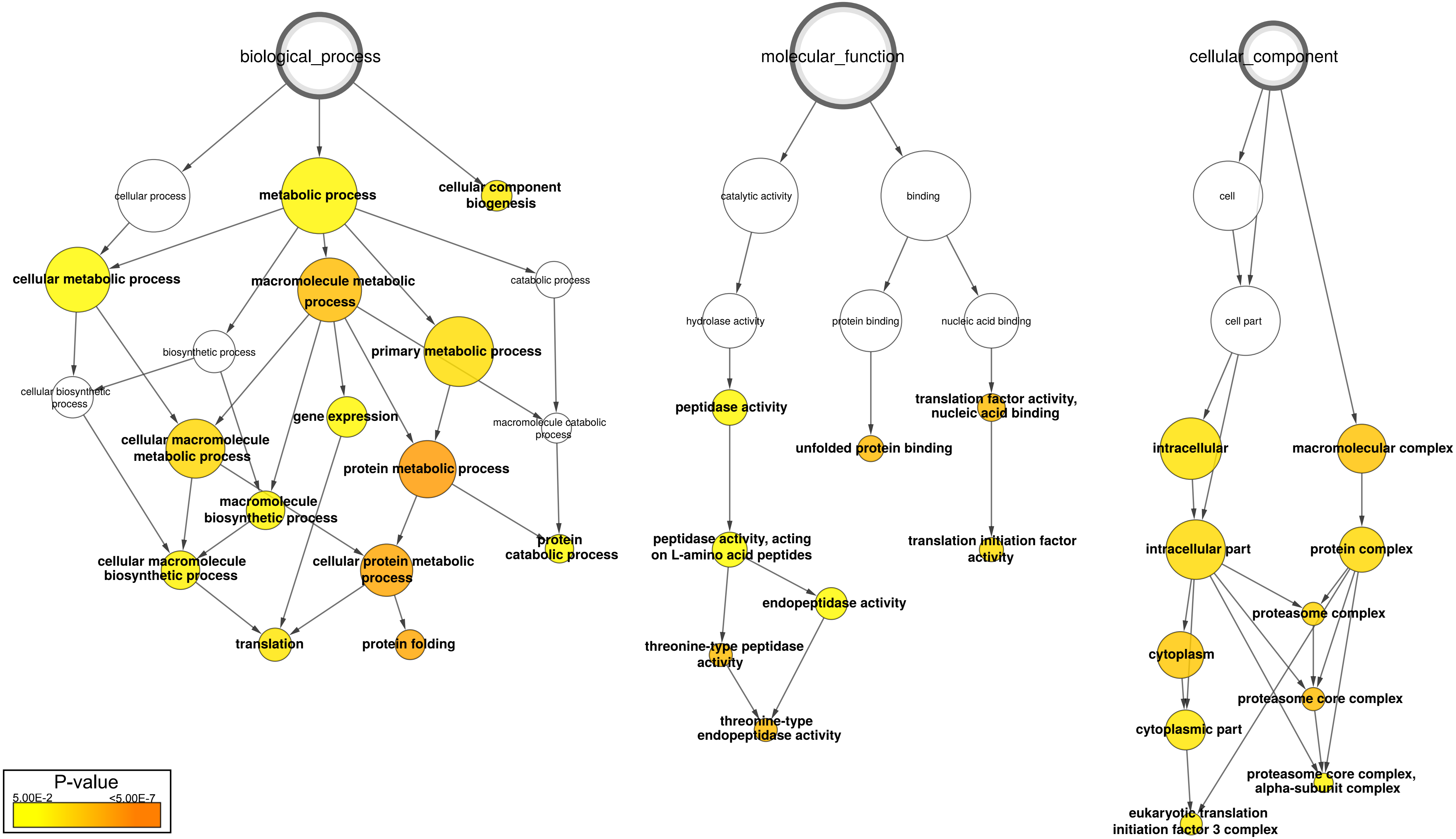

Figure S9: BiNGO GO enrichment (Biological Process; Molecular Function; Cellular Component) for the 'green' WGCNA module, which is part of the old subnetwork.
Colored nodes are GO terms that were significantly enriched after correcting for multiple testing (FDR).

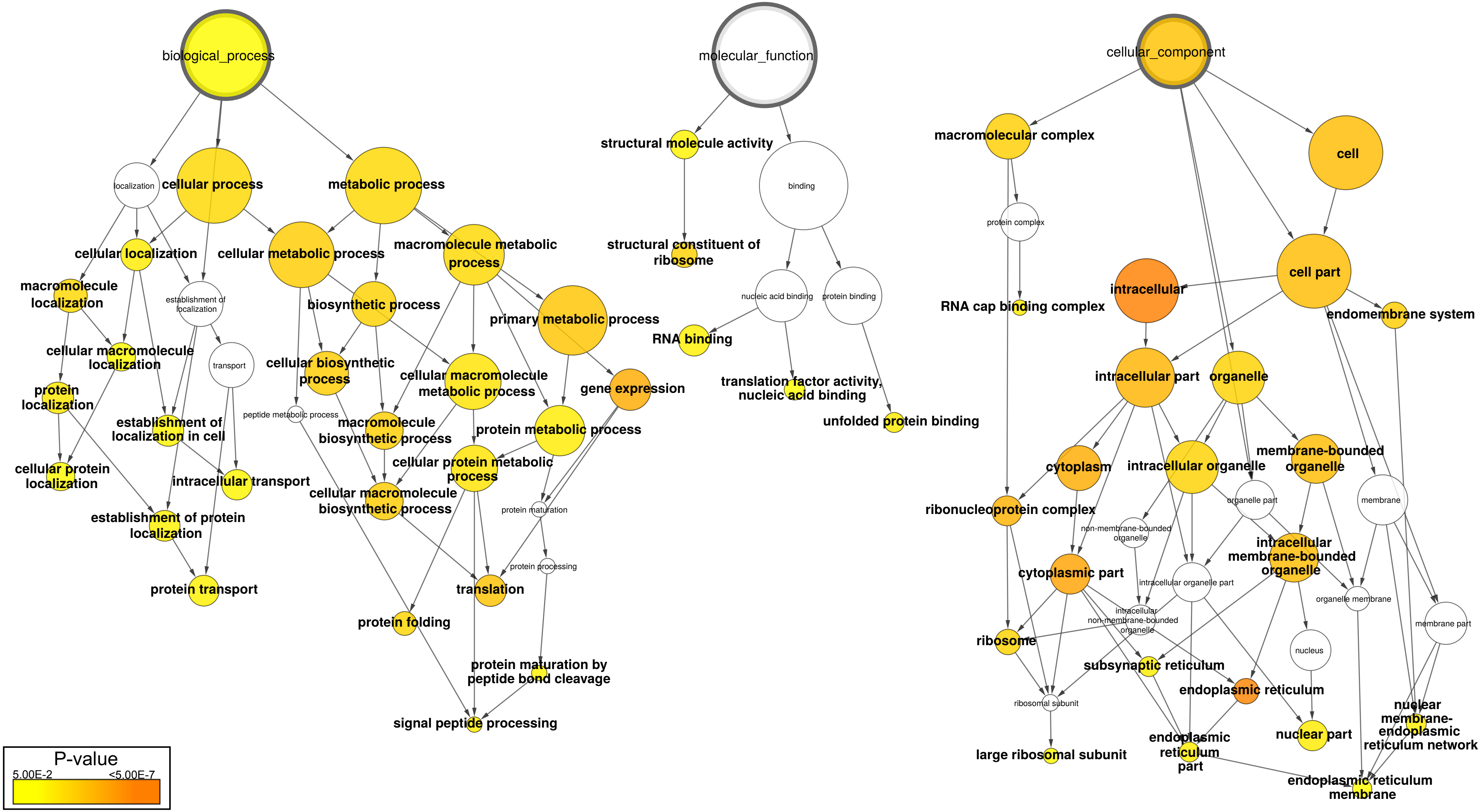

Figure S10: BiNGO GO enrichment (Biological Process, Molecular Function; Cellular Component) for the ‘paleturquoise’ WGCNA module, which is part of the old subnetwork.
Nodes in color are GO terms significantly enriched after correcting for multiple testing (FDR).
