# Supplementary material for: Disentangling the aging gene expression network of termite queens
Source: BMC Genomics. 2021 May 11;22:339. doi: 10.1186/s12864-021-07649-4 (PMC8114706; doi:10.1186/s12864-021-07649-4)

WGCNA module-age associations.

Listed are the eigengenes and the correlation coefficients for each with respect to age (p-values in parenthesis). Color code of the correlations range from 1 (red), meaning perfect positive correlation, over white (0: no correlation) to -1 (blue), meaning perfect negative correlation.

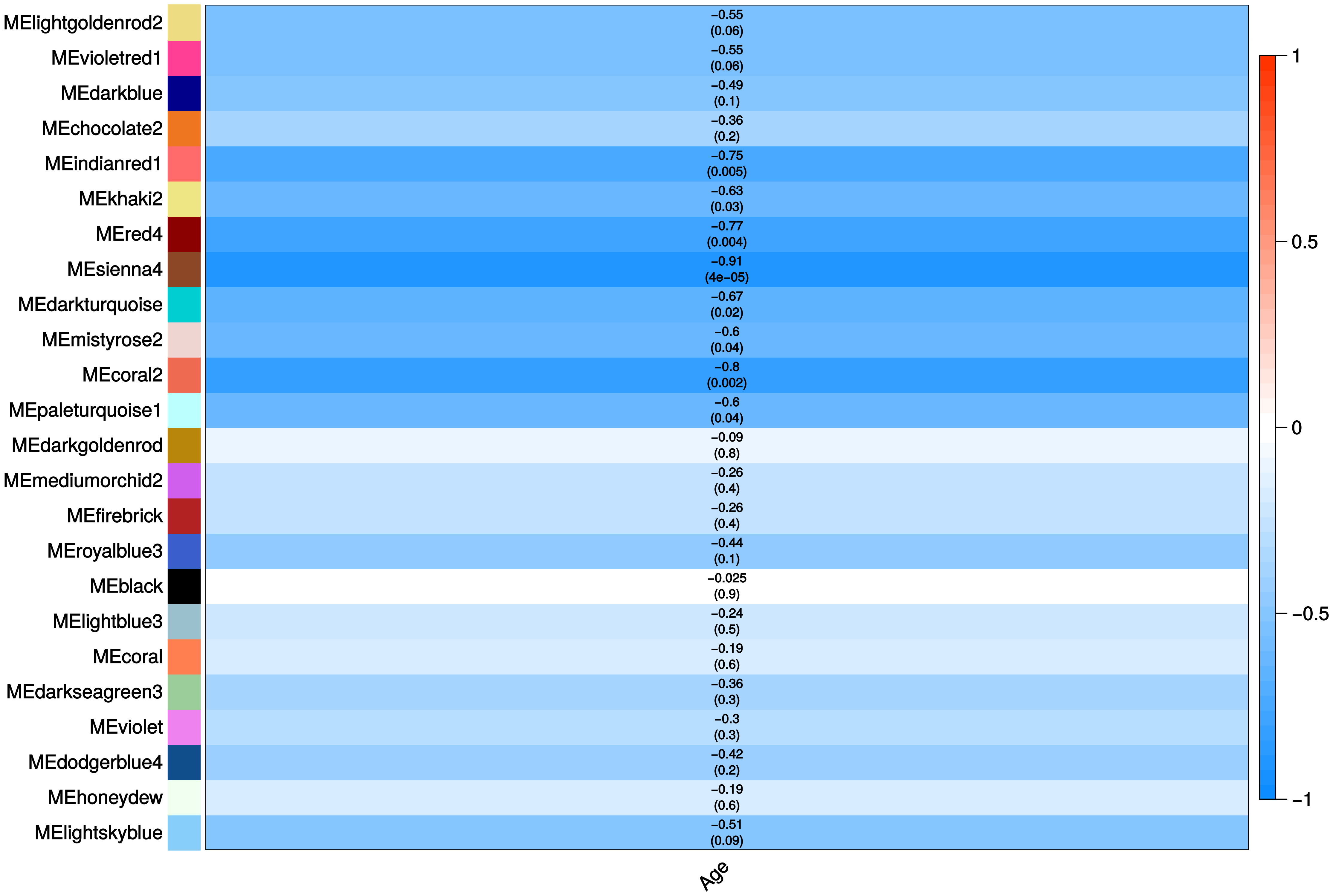

WGCNA module-age associations - continued.

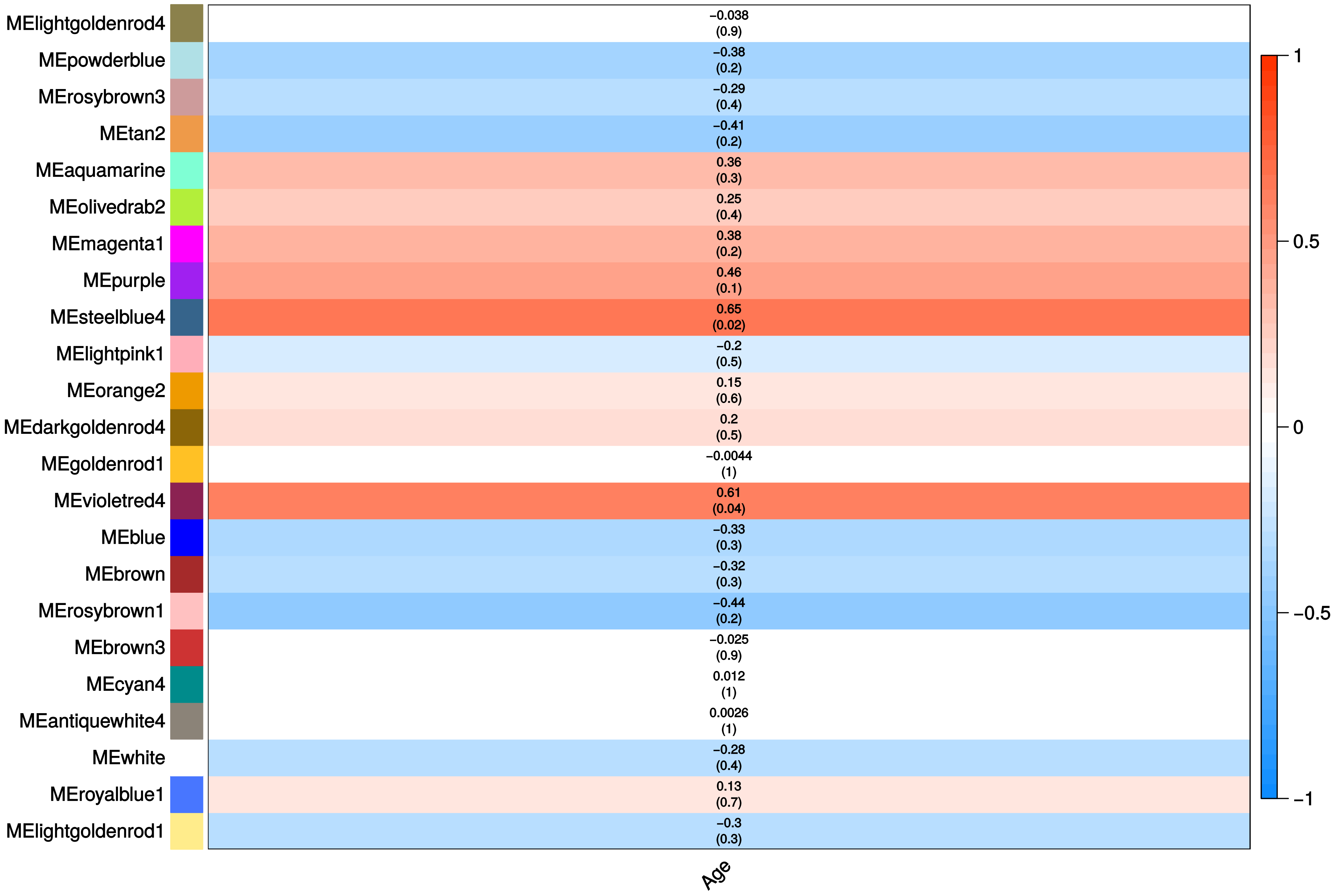

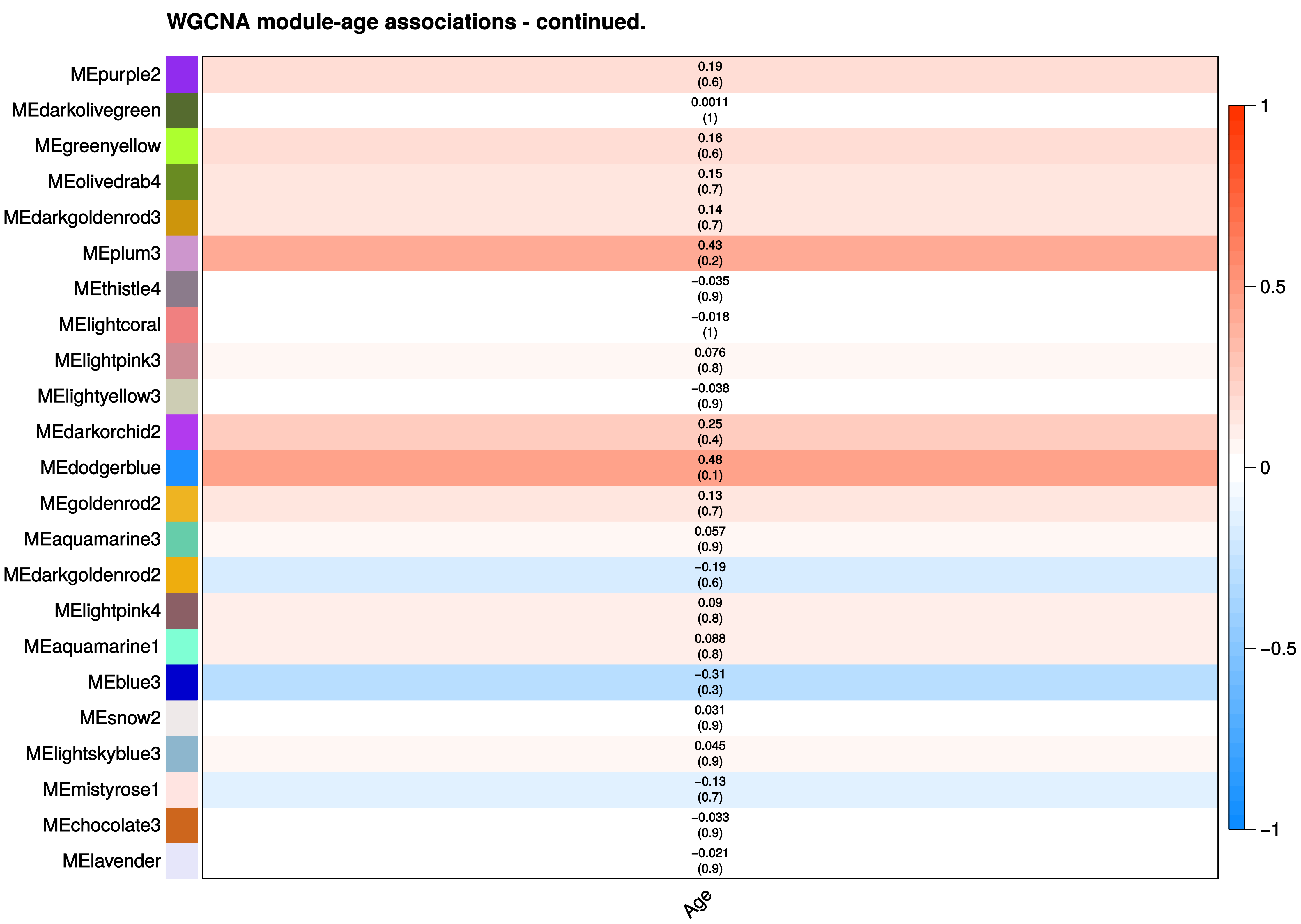
WGCNA module-age associations - continued.
MEpurple2
0.19
(0.6)
MEdarkolivegreen
0.0011
(1)
MEgreenyellow
0.16
(0.6)
MEolivedrab4
0.15
(0.7)
MEdarkgoldenrod3
0.14
(0.7)
MEplum3
0.43
(0.2)
MEthistle4
−0.035
(0.9)
MElightcoral
−0.018
(1)
MElightpink3
0.076
(0.8)
MElightyellow3
−0.038
(0.9)
MEdarkorchid2
0.25
(0.4)
MEdodgerblue
0.48
(0.1)
MEgoldenrod2
0.13
(0.7)
MEaquamarine3
0.057
(0.9)
MEdarkgoldenrod2
−0.19
(0.6)
MElightpink4
0.09
(0.8)
MEaquamarine1
0.088
(0.8)
MEblue3
−0.31
(0.3)
MEsnow2
0.031
(0.9)
MElightskyblue3
0.045
(0.9)
MEmistyrose1
−0.13
(0.7)
MEchocolate3
−0.033
(0.9)
MElavender
−0.021
(0.9)
Age
1
0.5
0
−0.5
−1

WGCNA module-age associations - continued.

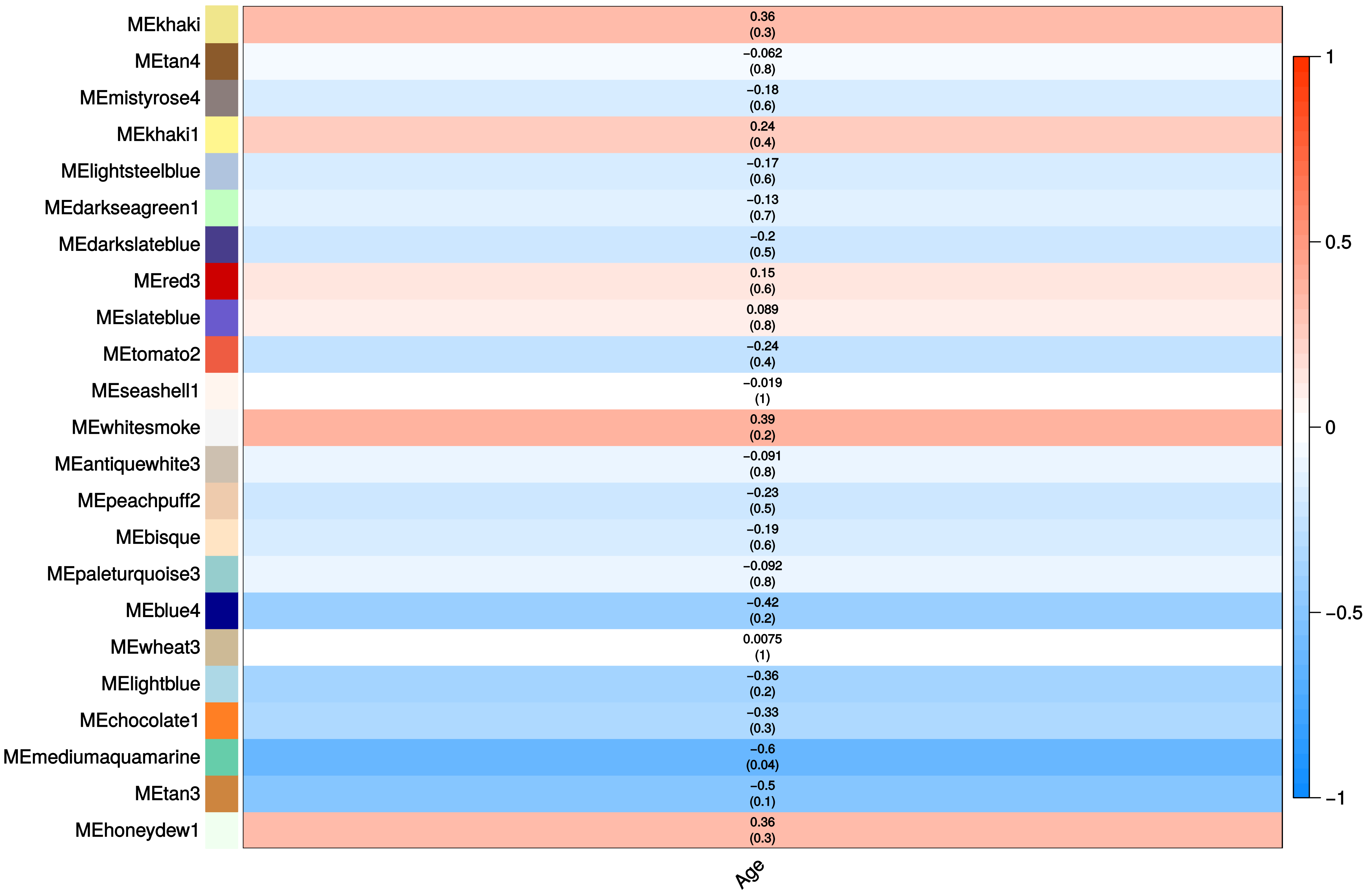

WGCNA module-age associations - continued.

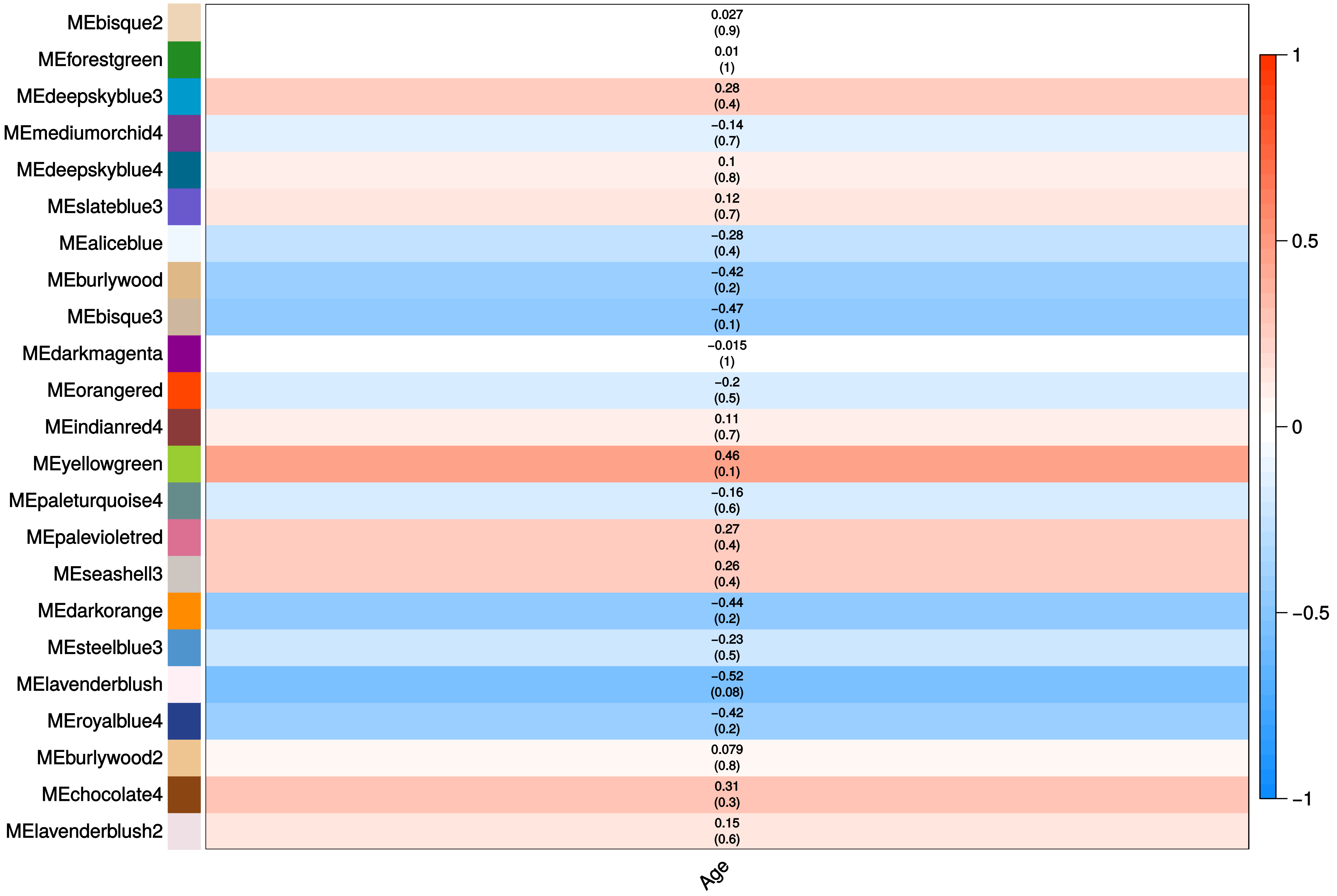

WGCNA module-age associations - continued.

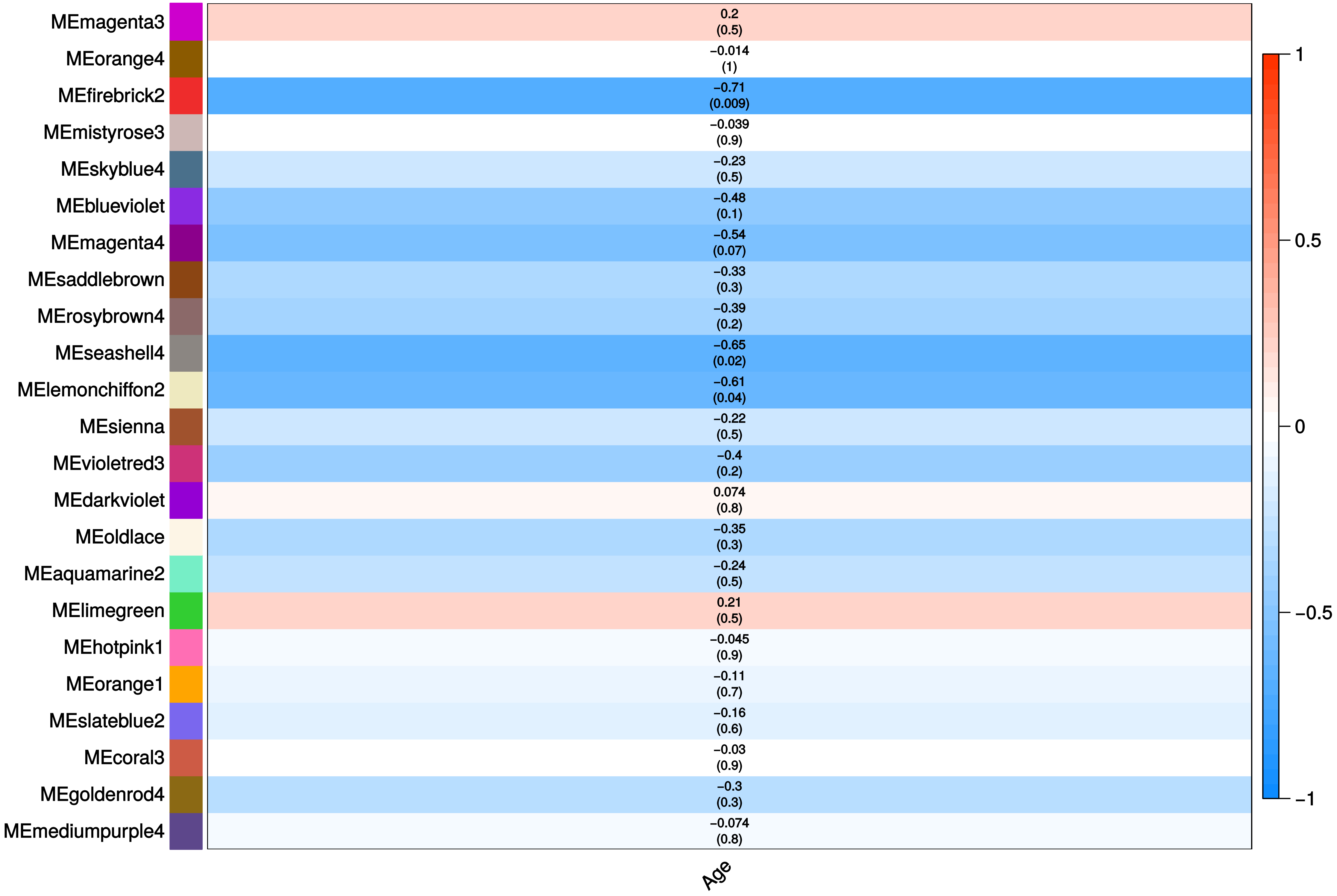

WGCNA module-age associations - continued.

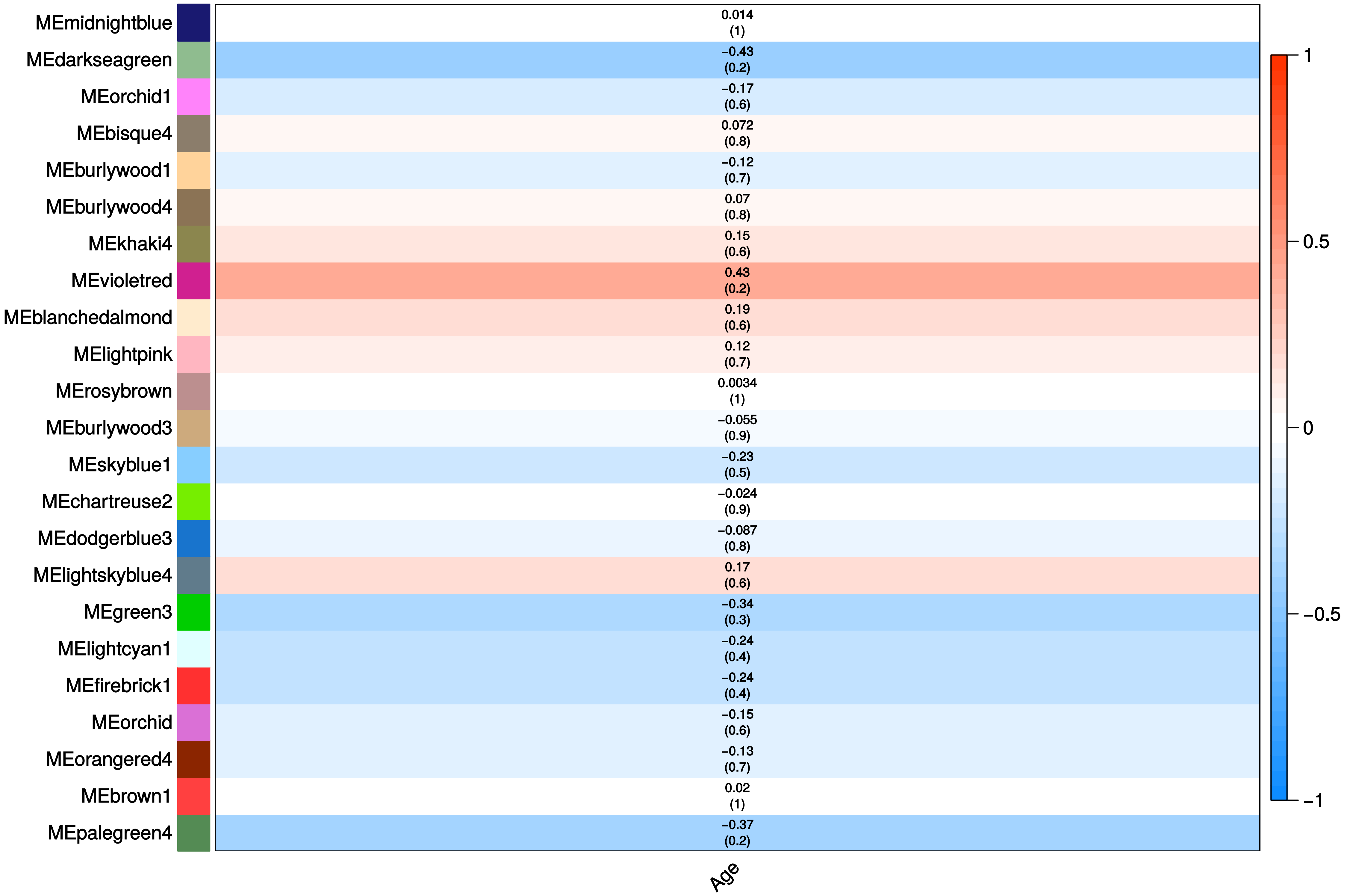

WGCNA module-age associations - continued.

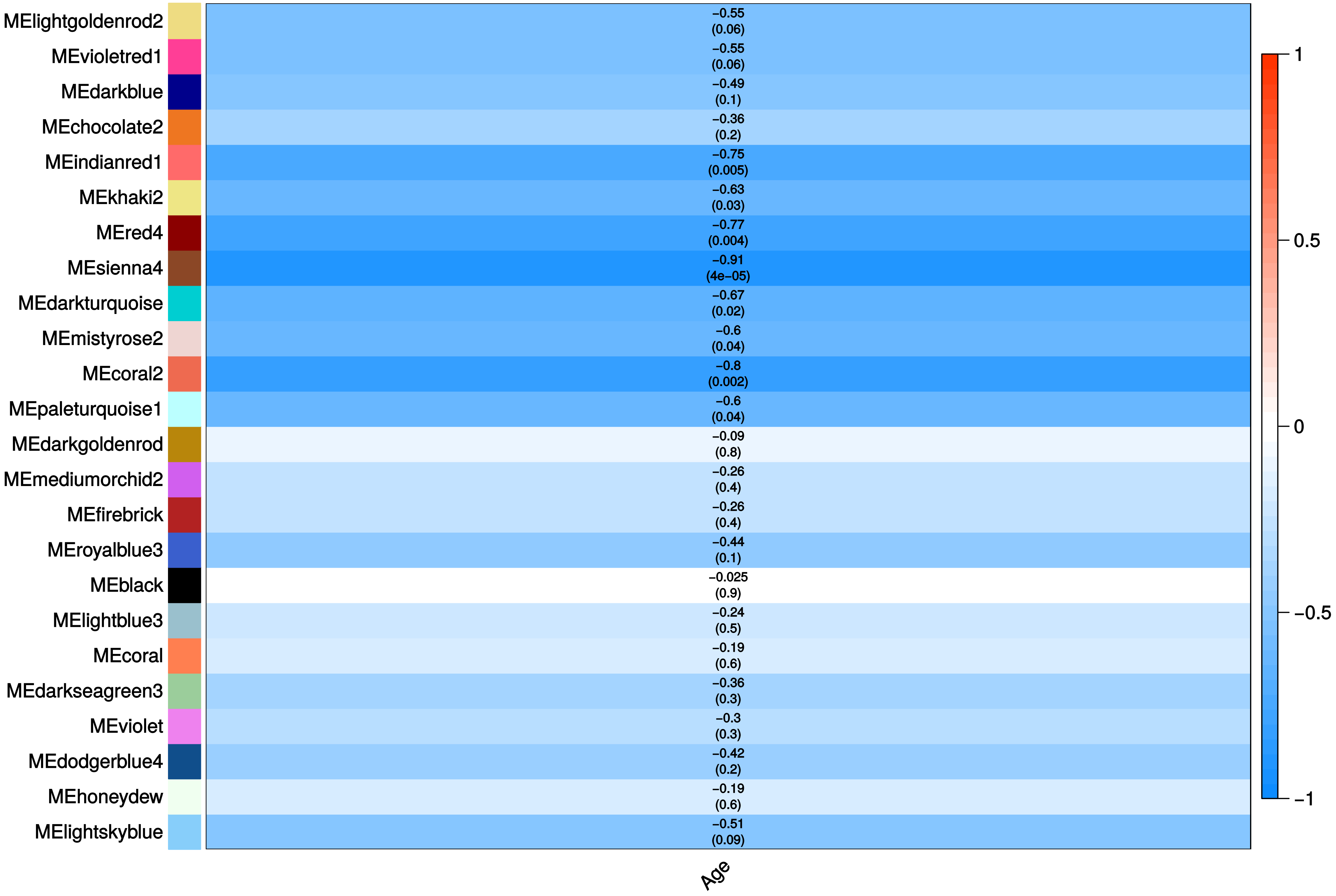

WGCNA module-age associations - continued.

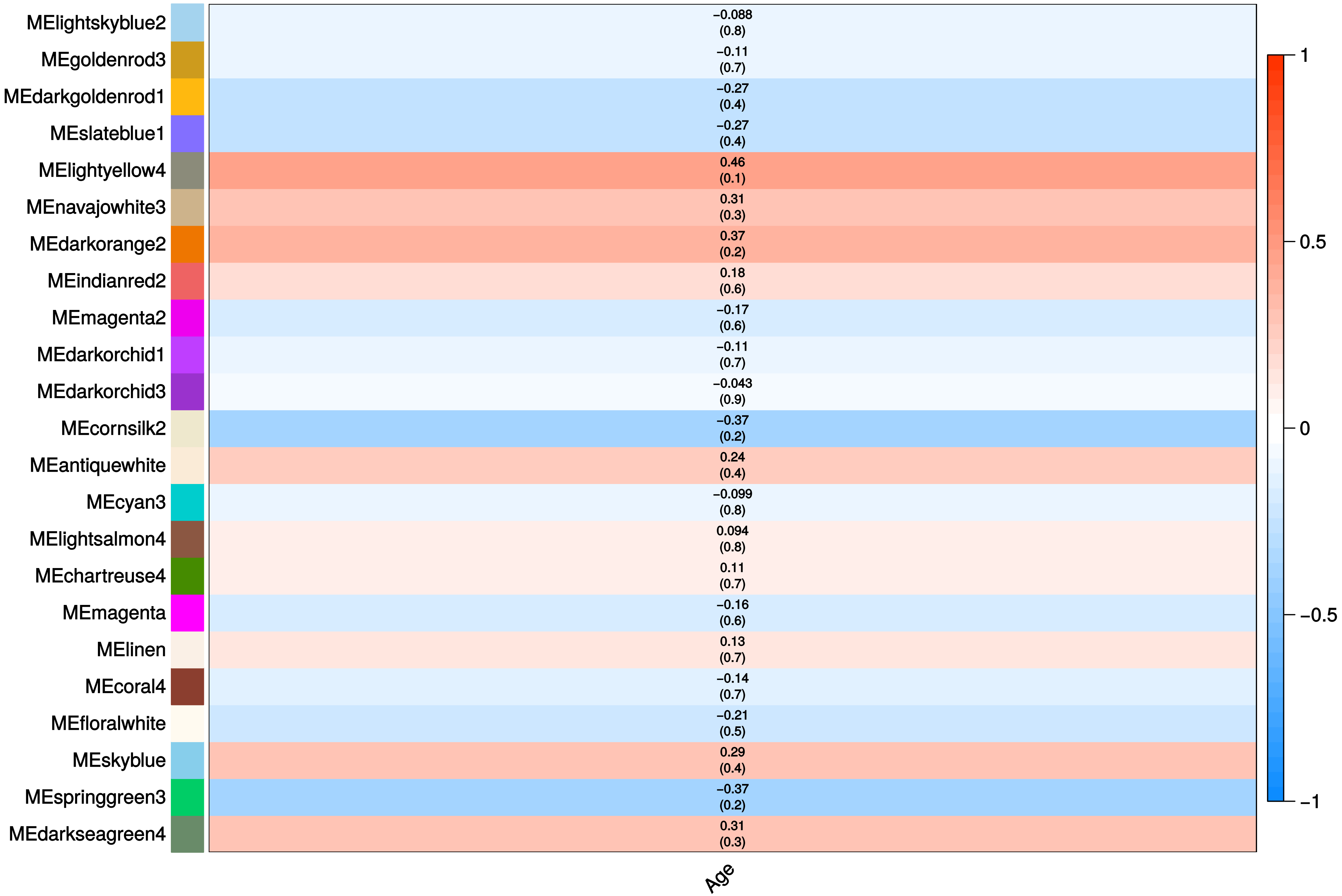

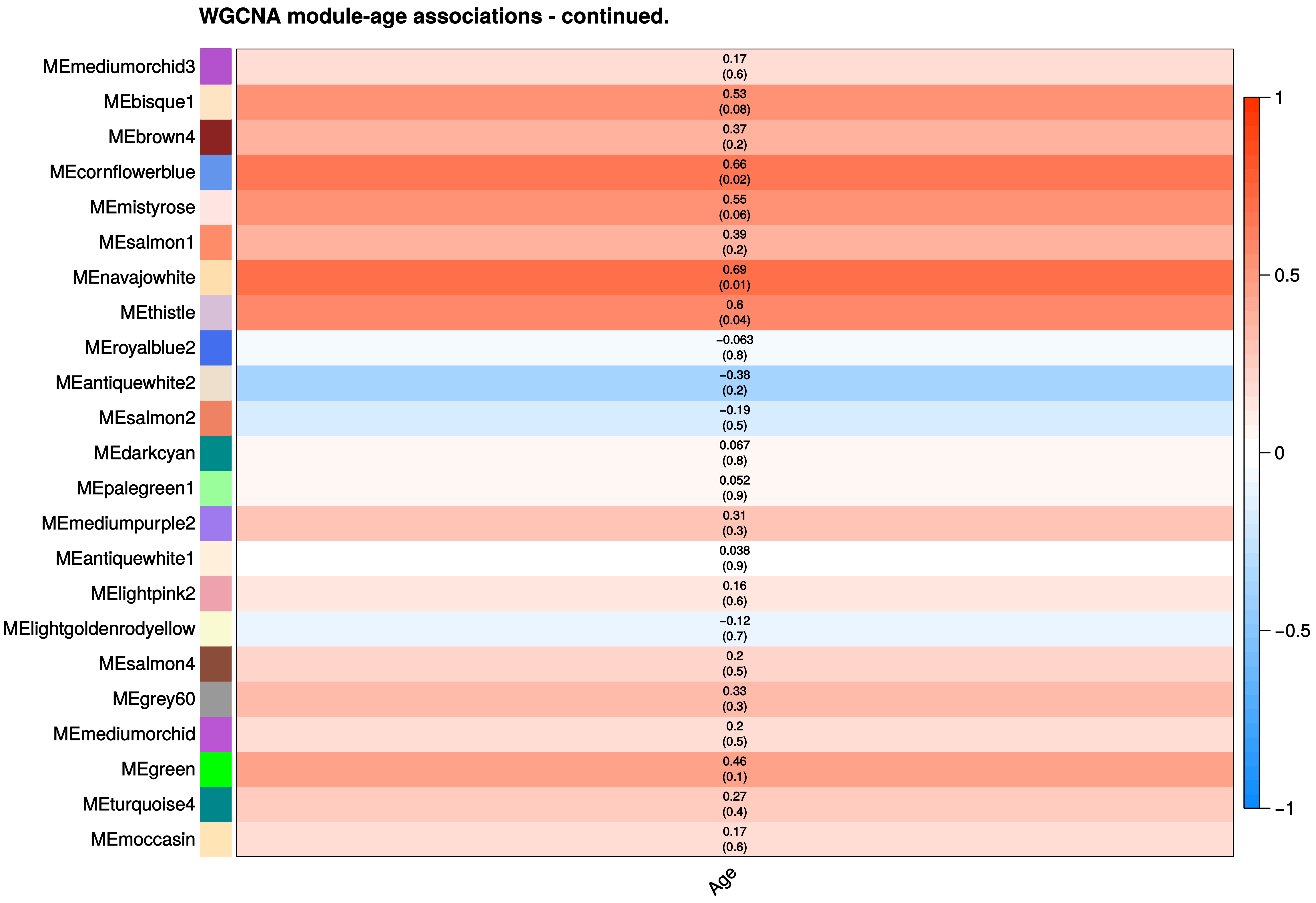
WGCNA module-age associations - continued.
MEmediumorchid3
0.17
(0.6)
MEbisque1
0.53
(0.08)
MEbrown4
0.37
(0.2)
MEcornflowerblue
0.66
(0.02)
MEmistyrose
0.55
(0.06)
MEsalmon1
0.39
(0.2)
MEnavajowhite
0.69
(0.01)
MEthistle
0.6
(0.04)
MEroyalblue2
−0.063
(0.8)
MEantiquewhite2
−0.38
(0.2)
MEsalmon2
−0.19
(0.5)
MEdarkcyan
0.067
(0.8)
MEpalegreen1
0.052
(0.9)
MEmediumpurple2
0.31
(0.3)
MEantiquewhite1
0.038
(0.9)
MElightpink2
0.16
(0.6)
MElightgoldenrodyellow
−0.12
(0.7)
MEsalmon4
0.2
(0.5)
MEgrey60
0.33
(0.3)
MEmediumorchid
0.2
(0.5)
MEgreen
0.46
(0.1)
MEturquoise4
0.27
(0.4)
MEmoccasin
0.17
(0.6)
Age
1
0.5
0
−0.5
−1

WGCNA module-age associations - continued.

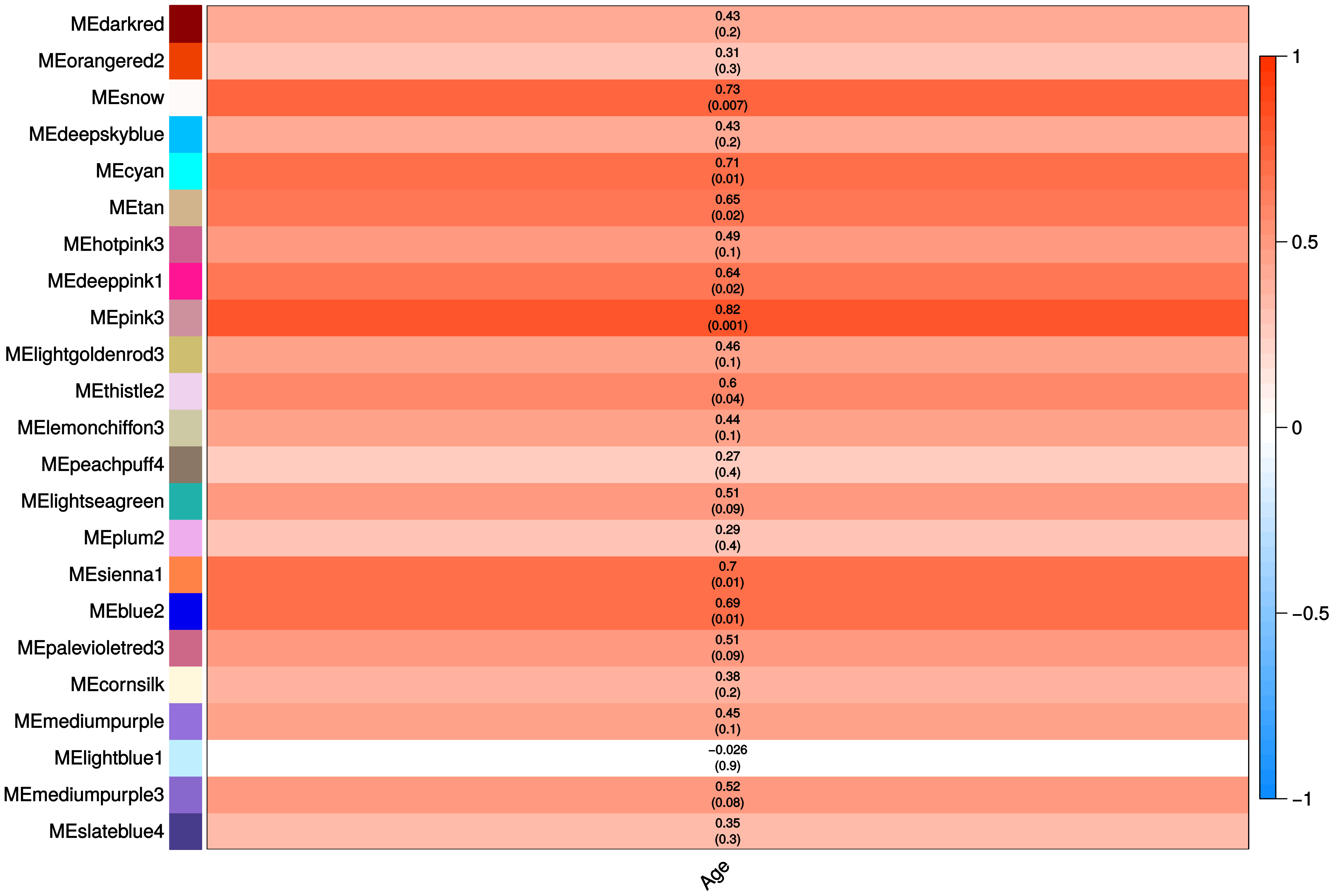

Supplement: Supplementary file 3 — Additional file 3. WGCNA module-age associations. Listed are the eigengenes and the correlation coefficients with respect to age (p-values in parenthesis). [file 12864_2021_7649_MOESM3_ESM.pdf]
